# Relationship between serum growth differentiation factor 15, fibroblast growth factor-23 and risk of atrial fibrillation: A systematic review and meta-analysis

**DOI:** 10.3389/fcvm.2022.899667

**Published:** 2022-08-04

**Authors:** Ziqi Tan, Tiangang Song, Shanshan Huang, Menglu Liu, Jianyong Ma, Jing Zhang, Peng Yu, Xiao Liu

**Affiliations:** ^1^Department of Endocrine, The Second Affiliated Hospital of Nanchang University, Nanchang, China; ^2^Department of Cardiology, Seventh People's Hospital of Zhengzhou, Henan, China; ^3^Department of Pharmacology and Systems Physiology University of Cincinnati College of Medicine, Cincinnati, OH, United States; ^4^Department of Anesthesiology, The Second Affiliated Hospital of Nanchang University, Nanchang, China; ^5^Department of Cardiology, The Second Affiliated Hospital of Nanchang University, Nanchang, China

**Keywords:** atrial fibrillation, biomarker, arrhythmia, GDF-15, FGF-23

## Abstract

**Background and objective:**

Growth differentiation factor-15 (GDF-15) and fibroblast growth factor-23 (FGF-23) are considered predictors of the incidence of cardiovascular diseases. The present meta-analysis aimed to elucidate the associations between GDF-15 and FGF-23 in the risk of atrial fibrillation (AF).

**Methods:**

An electronic search was conducted in the Cochrane Library, PubMed, and Embase databases from inception until February 27, 2021. The study protocol was registered in the PROSPERO database (CRD42020182226).

**Results:**

In total, 15 studies that enrolled 36,017 participants were included. Both serum FGF-23 and GDF-15 were elevated in patients with AF. Analysis of categorical variables showed higher serum FGF-23 levels were associated with an increased risk of AF [relative risk (RR) = 1.28, 95% confidence interval (CI): 1.05–1.56]. In contrast, this association was not found with GDF-15 (RR = 0.91, 95% CI: 0.20–4.04). In dose-response analysis, a linear positive association was noted between serum FGF-23 levels and the risk of AF (P _nonlinear_ = 0.9507), with a RR elevation of 7% for every 20 pg/ml increase in the serum FGF-23 levels (95% CI: 1.02–1.13). No remarkable relationship was found between serum GDF-15 levels and the risk of AF, and the overall RR for the association between a 100 ng/L increment in GDF-15 levels and AF was 1.01 (95% CI: 0.998–1.02).

**Conclusion:**

Our study showed a positive linear correlation between serum FGF-23 levels and the risk of AF. However, no significant association was found between GDF-15 and the risk of AF. Further studies are warranted to clarify whether serum FGF-23 levels may be considered in predicting the risk of AF.

**Systematic Review Registration:** http:www.york.ac.uk/inst/crd, identifier CRD42020182226.

## Introduction

Atrial fibrillation (AF) is the most prevalent sustained arrhythmia in clinical practice and is associated with high morbidity and mortality (1, 2). Although several traditional clinical risk factors such as hypertension, age, and metabolic syndrome were identified, the potentially modifiable risk factors for AF remain to be elucidated (3, 4). In recent years, several biomarkers have shown a strong association with the incidence and development of cardiovascular diseases, such as myocardial infarction, heart failure, and AF (5–7). Among them, growth/differentiation factor-15 (GDF-15) and fibroblast growth factor-23 (FGF-23) have been comprehensively investigated (8).

FGF-23 is a bone-derived hormone that plays an essential role in regulating the metabolism of phosphate and 1,25-dihydroxyvitamin D (9). In addition, it inhibits the renal synthesis of calcitriol and the secretion of parathyroid hormone from the parathyroid glands (9). Furthermore, higher FGF23 levels are linked with an increased risk of cardiovascular mortality (8, 10). GDF-15 is a growth factor that belongs to the transforming growth factor-β family. The expression of GDF15 rapidly increases in response to oxidative stress, myocardial stretch, volume overload, and myocardial inflammation (11). The expression levels of GDF-15 and FGF-23 have been shown to be associated with the prognosis of severe cardiovascular diseases, such as heart failure and AF (12, 13). Moreover, these markers may be closely correlated with an increased risk of AF in the general population (9, 12, 14–16). Conversely, several cohorts have reported a null association (17). Therefore, this study aims to assess the relationship between baseline GDF-15/FGF-23 levels and the risk of AF, and the potential dose-dependent effects.

## Methods

This study was conducted following the guidelines of the Preferred Reporting Item for Systematic Review and Meta-Analysis (PRISMA) (Supplementary Table 1). Additionally, this study was registered with PROSPERO (International prospective register of systematic reviews. http:www.york.ac.uk/inst/crd)-registration number-CRD42020182226.

### Literature search

The PubMed database, Embase database, and Cochrane database were searched using the following keywords up to February 27, 2021, with no language restriction. The search terms according to PICOS were as follows:

Exposure:

For GDF-15: “growth differentiation factor 15” OR “macrophage inhibitory cytokine 1” OR “prostate differentiation factor” OR “GDF-15”.
For FGF-23: “fibroblast growth factor-23” OR “FGF-23 protein” OR “fibroblast growth factor 23” OR “FGF-23 protein” OR “phosphatonin” OR “tumor-derived hypophosphatemia inducing factor”.

Outcomes:

For AF: “atrial fibrillation” OR “atrial flutter” OR “atrial arrhythmia” OR “atrial tachycardia”.
The detailed description of the search strategy was described in Supplementary Table 2.

### Study selection

The Endnote X9 (Thomson Reuters, New York, NY) database was used to manage all citations. The abstracts of the studies investigating the association between GDF-15 and FGF-23 were reviewed and the full texts were then searched.

The inclusion criteria were: (1) The article reported serum GDF-15/FGF-23 levels in the AF and non-AF populations; (2) Studies designed as observational studies (cohort, nest-control, or case-control) reported the association between baseline serum GDF-15/FGF-23 level and risk of AF, with adjusted odds ratios (OR), relative risk (RR) or hazard ratio (HR), and the corresponding 95% confidence interval (CI), or providing data to calculate these effects size. The exclusion criteria were: (1) articles with incomplete data provided, such as letters, comments, and reviews; (2) the cross-sectional studies were excluded due to the high risk for bias; (3) articles involved specific genetic polymorphisms; (4) AF were expressed at tissue or cell level, such as the degree of structural remodeling.

If the same population was used in multiple studies, the most informative article was included.

### Data extraction and quality assessment

According to the above inclusion criteria, the researchers (Z.Q-T and X-L) independently evaluated the eligibility of the literature. The basic characteristics of each study were extracted, including the first author, year of publication, age, gender, complications, sample size, adjusted estimated effect, 95% confidence interval of each category, and adjustments. The Newcastle-Ottawa Scale (NOS) was used for quality assessment of the articles, with scores ranging from 0 to 9. A higher grade (≥7) indicates a moderate-high quality; otherwise, the articles were regarded as low-quality (18, 19).

### Statistical analysis and bias risk assessment

The researchers converted the effect measure into its natural logarithm (RR) and calculated the standard error [selog (RR)] according to the corresponding 95% CI. Random-effects models were used considering the potential heterogeneity across studies. GDF-15 and FGF-23 levels were converted into a uniform unit across all included studies (pg/ml for FGF-23, ng/ml for GDF-15). To compare the GDF-15 levels between the AF and control groups, the GDF-15 and FGF-23 levels that were originally expressed as quartiles and medians were converted to mean and standard deviation (20, 21). The standardized mean difference (SMD) in GDF-15/FGF-23 between those with AF and those without AF was calculated. The SMD represents the difference between the weighted mean and SD of the GDF-15/FGF-23 of individuals with AF compared to the controls. In the linear exposure-effect analysis, the method described by Greenland and Longnecker (22) was used to estimate study-specific slopes and 95% CIs. The robust error meta-regression method developed by Xu and Doi (23, 24) was applied for the non-linear dose-response analysis. The levels of GDF-15 and FGF-23 and their effect size with variance estimates were required for at least two quantitative exposure categories. If the median or average level was not provided in the article, the average of the lower and upper limits of each category were used to estimate the midpoint. If the terminal category was open, the length of the open interval was assumed to be the same as that of the adjacent interval (25, 26). We applied I^2^ statistics to estimate the heterogeneity between studies. Low heterogeneity, moderate heterogeneity, and high heterogeneity were defined as *I*^2^ <50%, 50–75%, >75%, respectively (27). Review Manager (RevMan) version 5.4.1 (The Cochrane Collaboration 2014; Nordic Cochrane Center Copenhagen, Denmark) and STATA (Version 16.0, Stata Corp LP, College Station, Texas, USA) software were used for statistical analysis. Two-tailed *P* < 0.05 was considered statistically significant. In addition, to study the possible factors influencing the results, subgroup analysis was stratified by study design and adjustments (sex, NT-pro BNP and CRP).

## Results

### Study selection

**Figure 1:**
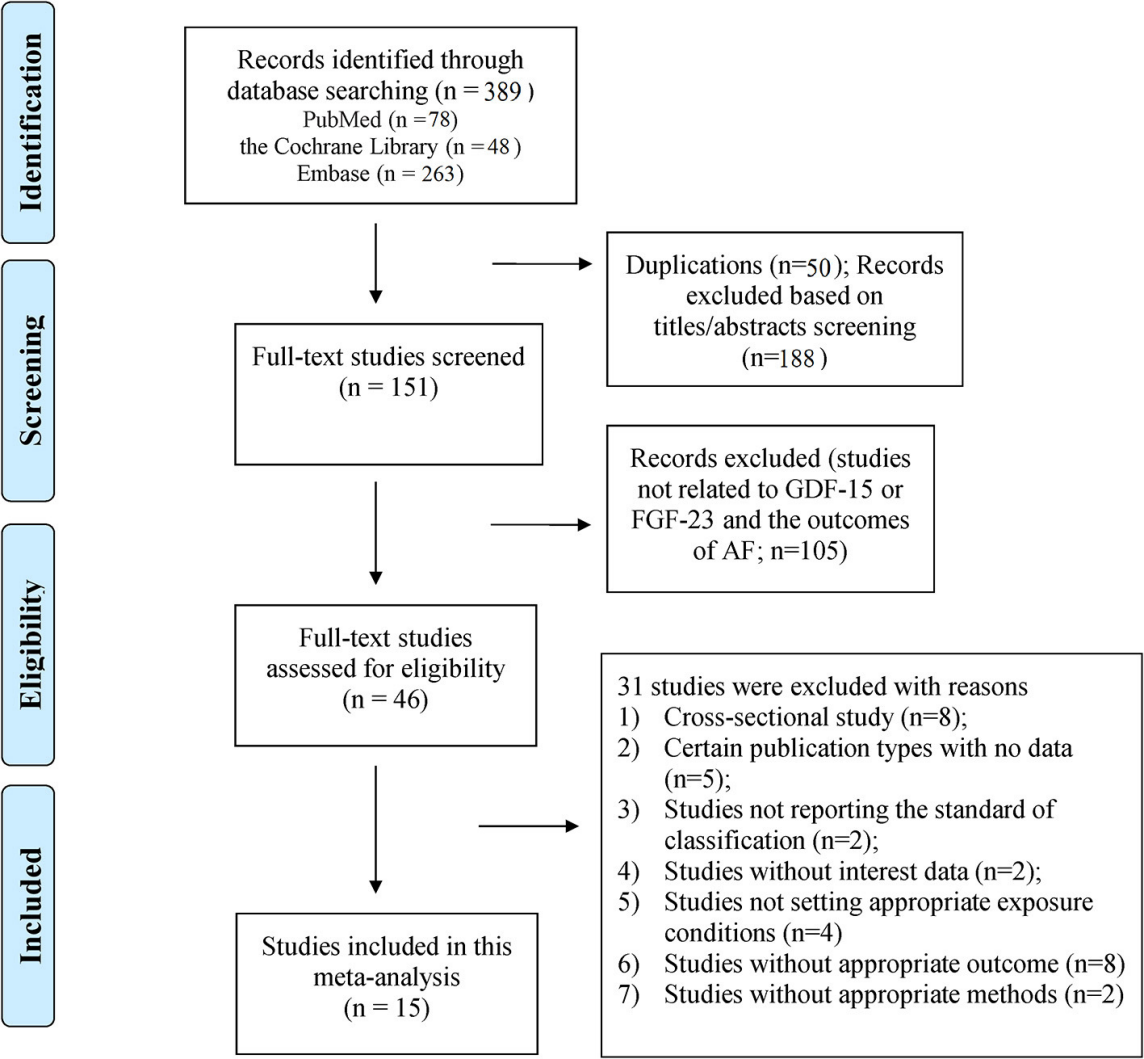
Flowchart of the study selection.

A total of 389 publications were initially retrieved (PubMed = 78; the Cochrane Library = 48; and Embase = 263). After removing 50 duplicates and 188 irrelevant citations, the full text of the remaining 151 articles were reviewed, and 15 studies (9 for GDF-15 and 6 for FGF-23) were finally included. The flowchart of the study selection is shown in Figure 1. The excluded studies (*n* = 31) are summarized with detailed reasons in Supplementary Table 3.

### Study characteristics and quality of the eligible studies

Table 1 shows the characteristics of the eligible studies. For GDF-15, 9 studies with 1,721 cases/10,602 individuals were included. In general, the eligible studies were published from 2011 to 2020, and their sample size ranged from 100 to 3,217 participants. Four studies reported the association between serum GDF-15 levels and the risk of AF in the general population (12–14, 29); 2 studies concentrated on patients who received coronary artery bypass graft (17, 28); and 2 studies reported this association in patients with recurrent AF after catheter ablation (30, 34). The majority of the eligible studies were performed in Europe (12, 15, 17, 28, 29, 34) (*n* = 6), two studies were undertaken in China (13, 30), and only one study was conducted in the United States (14).

**Table 1:** Basic characteristics of the articles included in the meta-analysis of GDF 15, FGF-23 and risk of atrial fibrillation.

**References**	**Country**	**Study design/mean follow-up time**	**Study populations**	**Cases/ sample size**	**Mean age/male**	**Baseline comorbidities** **(%)**	**AF diagnosis**	**Outcome report**	**Adjustment for covariates**
Bening et al. (17)	Germany	Prospective cohort/NA	NA postoperative atrial fibrillation	38/229	68.45/83.41%	NA	ECG	Difference	NA
Bouchot et al. (28)	France	Prospective cohort/1 year	University Hospital of Dijon Postoperative atrial fibrillation	34/100	64.02/92.00%	Hypertension: 64.0 Diabetes: 36.0	ECG	Difference Risk of AF	Age, the EuroSCORE (age, cardiac systolic function, cardiovascular risk factors) and left atrial diameter.
Lamprea-Montealegre et al. (14)	USA	Prospective cohort/1 year	Chronic Renal Insufficiency Cohort study CKD patients	279/3053	NA/NA	CVD history: 28.0 HF history: 6.0 Diabetes: 48.0	ECG	Risk of AF	Age, sex, race, site, diabetes mellitus, cardiovascular disease, smoking, 24 h urinary protein, estimated glomerular filtration rate, systolic blood pressure, body mass index, low-density lipoprotein, high-density lipoprotein, angiotensin-converting enzyme inhibitor/angiotensin II receptor blockers, diuretics, β-blockers, phosphate, parathyroid hormone, FGF-23.
Rienstra et al. (15)	Netherlands	Retrospective cohort/10 years	Community-based Framingham Heart Study	242/3217	59.00/46.00%	Diabetes: 11.0 HF: 1.0 Myocardial infarction: 4.0	ECG	Risk of AF	Sex, age, smoking status, height, weight, systolic and diastolic blood pressure, hypertension treatment, diabetes status, heart failure, myocardial infarction, logeCRP and logeBNP.
Santema et al. (12)	Netherlands	Prospective cohort/NA	Six centers in Scotland	733/1758	72.50/72.53%	Diabetes history: 34.5; Stroke history: 10.4; Hypertension history: 68.9	ECG	Difference	NA
Shao et al. (13)	China	Prospective cohort/NA	Second Hospital of Tianjin Medical University	67/134	66.60/43.38%	Hypertension: 65.7 Diabetes: 13.4	NA	Difference Risk of AF	NA
Smit et al. (34)	Netherlands	Prospective cohort/1 year	University Medical Center Groningen AF recurrence	30/100	65.00/74.00%	Hypertension: 67.0 HF history: 20.0 Coronary artery disease: 18.0 Diabetes: 14.0	ECG	Difference	NA
Svennberg et al. (29)	Sweden	Prospective cohort/13 years	The Uppsala Longitudinal Study of Adult Men	113/883	71.00/100.00%	Diabetes: 10.3	ECG	Difference	NA
		Prospective cohort/10 years	The Prospective Investigation of the Vasculature in Uppsala Seniors	148/978	70.00/49.00%	Diabets: 11.7			
Wei et al. (30)	China	Prospective cohort/14 months	Peking University third hospital Postoperative atrial fibrillation	37/150	64.00/56.76%	Hypertension 62.7 Diabetes: 23.3 Coronary artery diseas: 12.7 Chronic HF: 6.7	ECG	Difference Risk of AF	Age, persistent AF, diabetes mellitus, NT-proBNP, eGFR, LAD, LAAV, ablative strategy (CPVI-only).
Alonso et al. (9)	USA	Retrospective cohort/17 years	Atherosclerosis Risk in Communities study	1572/12349	NA/NA	Diabetes: 14.3	ECG	Risk of AF	Age, race, sex, study site, body mass index, smoking, education, height, diabetes, systolic and diastolic blood pressure, use of antihypertensive medication, prevalent coronary heart disease, prevalent heart failure, ECG-based left ventricular hypertrophy, NT-proBNP, high-sensitivity C-reactive protein, eGFR, serum calcium, phosphorus, parathyroid hormone and 25-hydroxyvitamin D.
Chen et al. (31)	China	Prospective cohort/NA	Dongguan Songshan Lake Central Hospital	240/390	60.01/68.21%	NA	ECG	Difference	NA
Maan et al. (32)	Greece	Retrospective cohor/10.6 years	Multi-Ethnic Study of Atherosclerosis study	77/983	59.68/43.03%	Diabetes: 11.2	ECG	Difference Risk of AF	Age, gender, current smoking status, Ln NT-proBNF, Ln IL-6.
Mathew et al. (8)	USA	Retrospective cohor/7.7 yearst	Multi-Ethnic Study of Atherosclerosis	291/6398	NA/46.73%	Diabetes: 12.3 Hypertension: 36.4	ECG and physician claims data	Risk of AF	Age, gender, race/ethnicity, study site, attained education, low density cholesterol, use of lipid-lowering medications, current smoking, diabetes, physical activity, height, height squared, weight, urine albumin-creatinine-ratio, estimated glomerular filtration rate, systolic blood pressure, use of hypertension medication, the serum concentrations of calcium, phosphate, 25-hydroxyvitamin D and parathyroid hormone, NT-proBNF.
		Retrospective cohort/8 years	Cardiovascular Health Study	229/1350	NA/28.67%	Diabetes: 10.6 Hypertension: 46.0			
Mehta et al. (10)	USA	Prospective cohort/7.6 years	Chronic Renal Insufficiency Cohort CKD patients	660/3876	57.66/55.21%	Hypertension: 86.1 Diabetes: 48.5 HF: 9.7 CVD: 13.5	ECG	Difference Risk of AF	Age, sex, race/ethnicity, cardiovascular disease, systolic blood pressure, diabetes, smoking, diuretic use, estimated glomerular filtration rate, ratio of urinary albumin to creatinine, levels of calcium, phosphate, and parathyroid hormone.
Mizia-Stec et al. (33)	Poland	Case-control study/NA	NA Postoperative atrial fibrillation	69/NA	56.59/66.70%	Coronary artery disease: 20.3 Hypertension: 59.4 Diabetes: 17.4	ECG	Difference	NA

NA, not applicable; SR, sinus rhythm; ECG, electrocardiograms; NT-proBNP, N-terminal pro-B-type natriuretic peptide; IL-6, interleukin 6; eGFR, estimated glomerular filtration rate; EF, ejection fractions; LV, left-ventricular; BB, beta-blockers; ACE-I, angiotensin-converting enzyme inhibitors; ECG, electrocardiogram; AF, Atrial fibrillation; NT-proBNP, N-Terminal pro-brain natriuretic peptide; eGFR, estimated glomerular filtration rate; LAD, left atrial diameter; LAAV, left atrial appendage flow velocity; CPVI, circumferential pulmonary vein isolation; CRP, C-reactive protein; BNP, B-type natriuretic peptide; FGF-23, fibroblast growth factor-23; GDF-15, Growth differentiation factor-15; EuroSCORE, European System for Cardiac Operative Risk Evaluation; HF, heart failure; CVD, cardiovascular disease; CKD, chronic kidney disease. Difference, comparison of serum GDF-15 or FGF-23 level between patients with and without AF.

Six studies enrolling 3,138 cases and 25,415 participants reported the association between serum FGF-23 levels and the risk of AF from 2014 to 2020 (8–10, 31–33). Four studies reported an association between FGF-23 and AF in the general population (8, 9, 31, 32), one study was based on patients with CKD (10), and one study reported an association between FGF-23 and postoperative atrial fibrillation (33). Among them, 3 studies were performed in the United States (8–10), and others were conducted in Asian (*n* = 1) (31) or European countries (*n* = 2) (32, 33).

Ascertainment of AF in most studies was mainly conducted through electrocardiography or medical records; Shao's study did not specify the method of AF diagnosis (13).

These studies achieved Newcastle-Ottawa Scale (NOS) scores >6 points, and their estimated quality was acceptable (Supplementary Table 4).

### GDF-15

#### Comparison of serum GDF-15 levels between patients with and without AF

**Figure 2:**
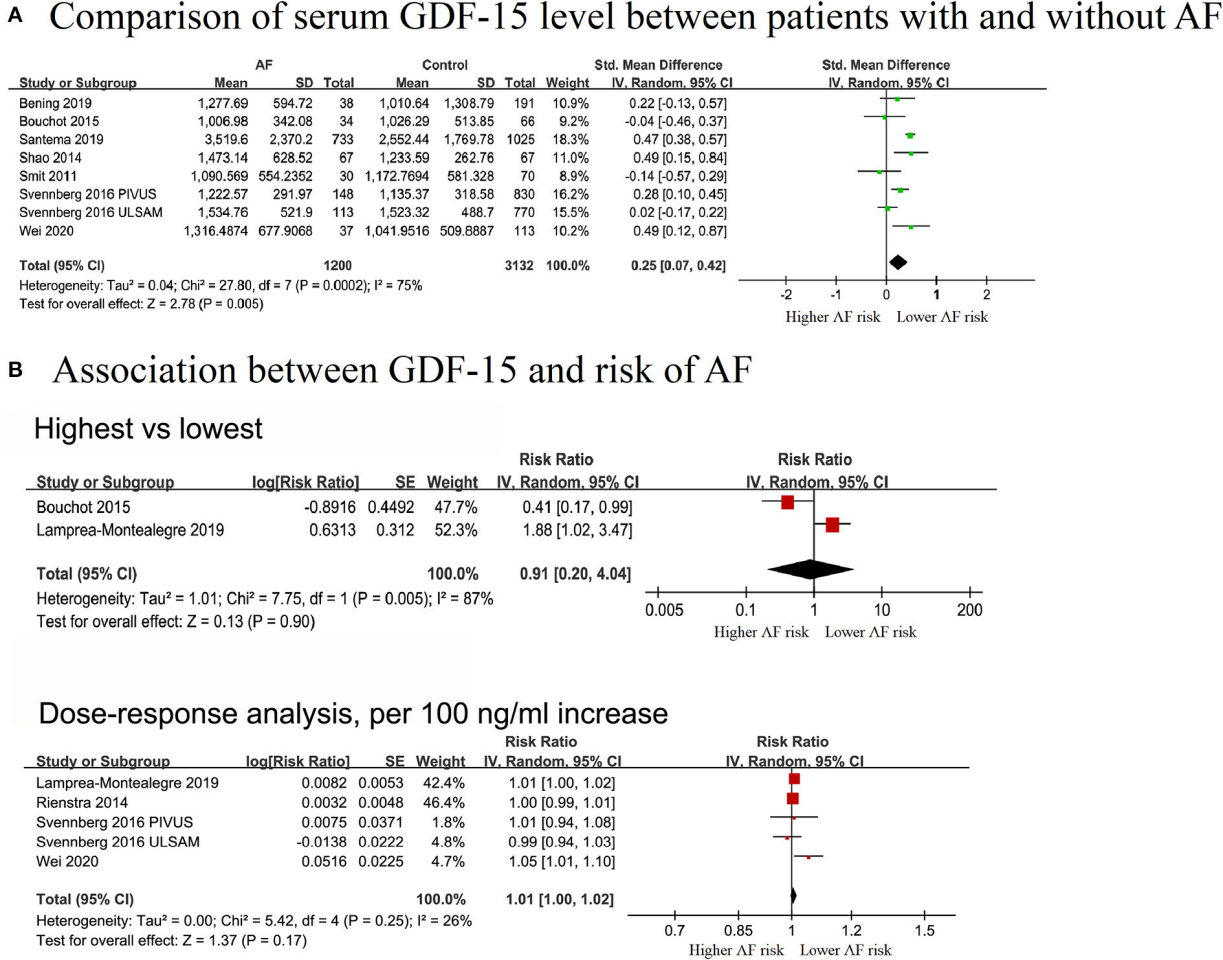
Forest plot showing the differences in serum growth differentiation factor 15 in controls without AF and patients with AF **(A)** and the association between serum growth differentiation factor 15 and atrial fibrillation **(B)**, upper panel: categorical analysis between growth differentiation factor 15 level and the risk of AF; lower panel: dose-response association between growth differentiation factor 15 and the risk of atrial fibrillation, per a 100 ng/ml increase.

A total of 7 studies with 1,200 cases/4,332 individuals were included (12, 13, 17, 28–30, 34). Serum GDF-15 levels were elevated in patients with AF [standardized mean difference (SMD): 0.25, 95% CI: 0.07–0.42; *I*^2^ = 75%] compared to patients without AF, with a significant heterogeneity (Figure 2A).

#### Association between GDF-15 and risk of AF

Two cohorts (313 cases and 3,153 individuals) were included in the categorical analysis (14, 28). The results showed that elevated GDF-15 levels were not significantly associated with a decreased risk of AF (RR = 0.91, 95% CI: 0.20–4.04; *I*^2^ = 87%), and significant heterogeneity was detected (Figure 2B).

In the dose-effect analysis, 5 cohorts from four publications (14, 15, 29, 30), covering 819 cases and 8,281 individuals, were included. The overall RR for assessing the association between a 100 ng/L increment in GDF-15 level and AF risk was 1.01 (95% CI: 0.998–1.02; *I*^2^ = 35%), with no evidence of heterogeneity (Figure 2B). The non-linear analysis was not performed due to limited data. In the pre-defined subgroup analyses stratified by study design, adjusted for gender, NT-pro BNP, and CRP, the results were still not significant. No significant subgroup differences were found among these groups (*P* > 0.05) (Supplementary Figures 1A–D).

### FGF-23

#### Comparison of FGF-23 levels between patients with and without AF

**Figure 3:**
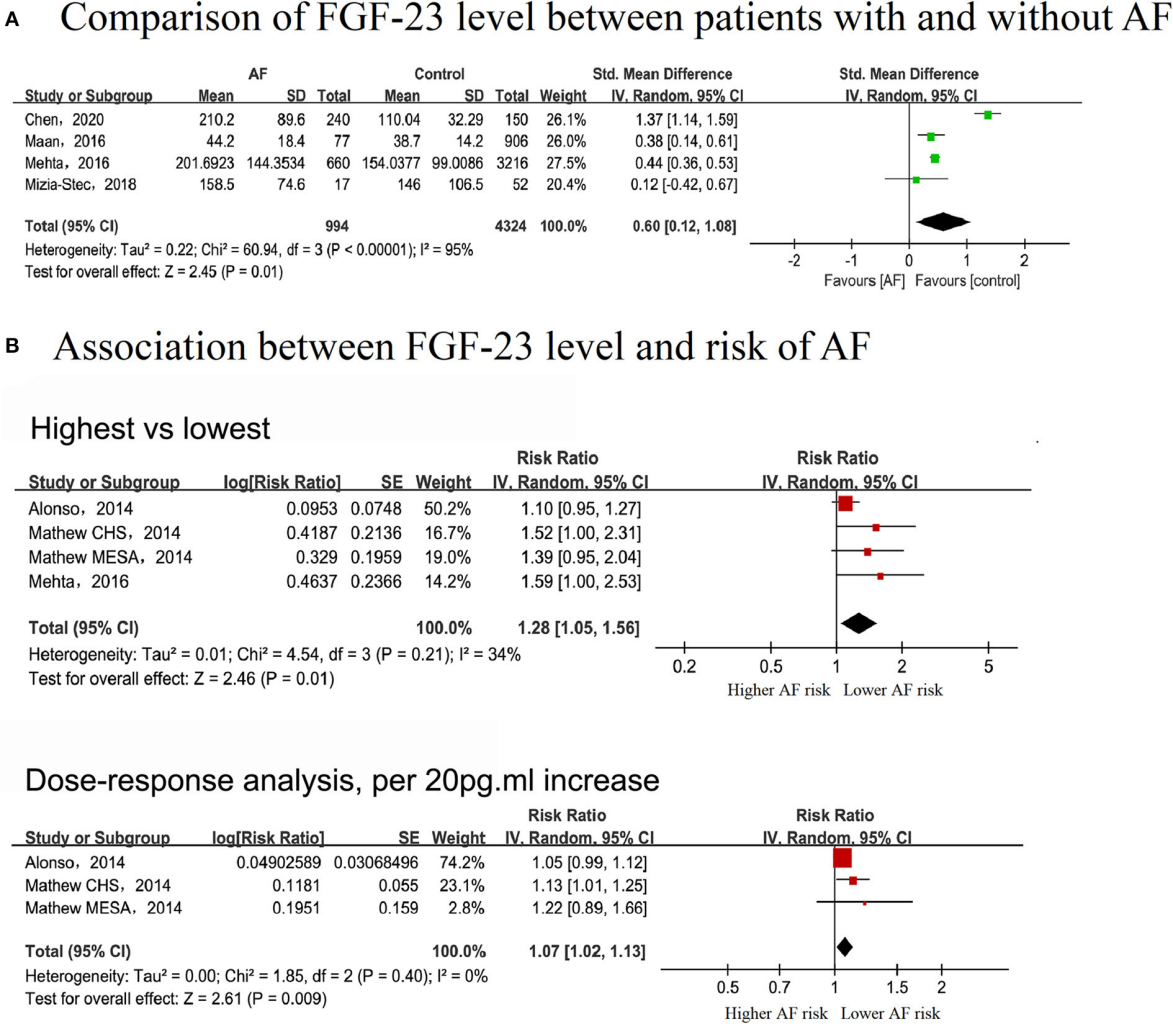
Forest plot showing the differences in serum fibroblast growth factor-23 level in controls without AF and patients with AF **(A)** and the association between serum fibroblast growth factor-23 level and the risk of AF **(B)**, upper panel: categorical analysis between fibroblast growth factor-23 level and the risk of AF; lower panel: dose-response association between fibroblast growth factor-23 level and the risk of AF, per 20 pg/ml increase.

Four studies (10, 31–33) that enrolled 994 cases and 5,318 individuals were included to explore the difference in FGF-23 levels between AF and non-AF patients. Patients with AF exhibited elevated serum FGF-23 levels (SMD: 0.55, 95% CI: 0.13–0.98; *I*^2^ = 94%), with substantial evidence of heterogeneity (Figure 3A).

#### Association between FGF-23 level and risk of AF

In the categorical analysis, three studies with 2,752 cases and 23,973 participants were included (8–10). The pooled RR for the correlation of serum FGF-23 level with AF risk was 1.28 (95% CI: 1.05–1.56, *I*^2^ = 34%), with no evidence of heterogeneity (Figure 3B). According to pre-defined subgroup analyses, the results were stable, and no subgroup differences were detected among these groups (*P* > 0.05) (Supplementary Figures 1E–G).

**Figure 4:**
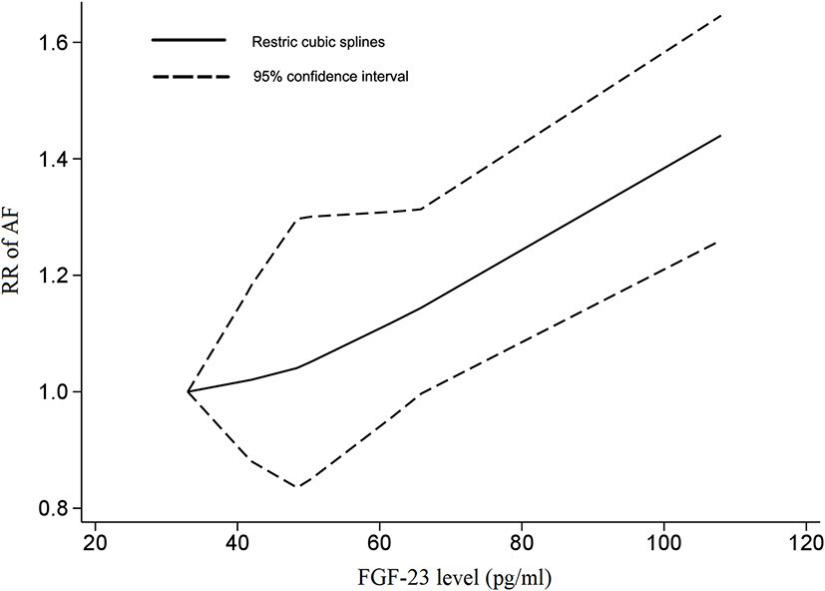
The dose-response association between the fibroblast growth factor-23 level and the risk of AF. In a non-linear exposure-effect analysis, the solid and dashed lines represent the estimated relative risk and the 95% confidence interval, respectively.

Three cohorts in two studies, covering 2,092 AF cases and 20,097 participants, were included in the dose-response analysis (8, 9). There was a linear correlation between serum FGF-23 levels and the risk of AF (P_non−linear_ = 0.9507), with an FGF-23 cutoff value of 62 pg/ml indicating a significantly increased risk of AF (Figure 4). The overall RR for the association between a 20 pg/ml increase in serum FGF-23 level with AF risk was 1.07 (95% CI: 1.02–1.13; *I*^2^ = 0%), with no evidence of heterogeneity (Figure 3B). All included studies were adjusted for gender and NT-pro BNP in the exposure-response analysis; thus, the dose-response analysis in subgroups stratified by gender and NT-pro BNP were not performed.

## Discussion

### Major findings

The present study showed that serum FGF-23 levels were linearly correlated with the risk of AF, with a RR increase of 7% for every 20 pg/ml elevation in the FGF-23 level. However, although AF patients had a higher serum GDF-15 level, a positive association between serum GDF-15 levels and the risk of AF was not established, either in the categorical or continuous variables analyses.

### Comparison with previous studies

#### GDF-15

The relationship between serum GDF-15 levels and the risk of AF remains inconclusive (14, 30). Importantly, although we found a noticeable increase in serum GDF-15 levels in patients with AF compared with those without AF, no positive association between GDF-15 and AF risk was found. This result was confirmed in the sensitivity and subgroup analyses. In addition, in a community-based Swedish study, a neutral association was reported between serum GDF-15 levels and the risk of AF [hazard ratio (HR): 1.141, *P* = 0.12] (35). Notably, GDF-15 levels can predict adverse outcomes (e.g., major bleeding) in patients with AF, rather than being markers for AF incidence among the general population. Moreover, the prognostic value of GDF-15 for other outcomes of AF patients, such as recurrence of AF after catheter ablation, was also reported in several studies (30, 36, 37). However, owing to the limited sample size, the association between serum GDF-15 levels and the risk of AF should be further evaluated.

#### FGF-23

Previous studies regarding the association between serum FGF-23 levels and the risk of AF yielded inconsistent results (8–10). A cross-sectional study of Japanese cardiac patients first reported a U-shaped relationship between serum FGF23 levels and the prevalence of AF (38). However, the ARIC study demonstrated an approximately linear correlation between serum FGF-23 levels and AF incidence (9). A meta-analysis showed a positive correlation of serum FGF-23 levels with the risk of AF, but only categorical variables were analyzed, and the potential dose-dependent effects were not evaluated (39). For the first time, the present study showed a positive linear correlation between serum FGF-23 levels with the risk of AF, with a 7% increased risk of AF for each 20 pg/ml elevation in serum FGF-23 levels. Notably, the relationship between serum FGF23 levels and AF incidence might be markedly influenced by kidney function. Alson et al. found a linear association in the overall population in the ARIC cohort. However, a U-shaped relationship was found for the subgroup with eGFR >60 mL/min per 1.73 m^2^, and an inverse U-shaped relationship was suggested for the subgroup with eGFR <60 mL/min per 1.73 m^2^ (9). FGF-23 is a well-known mediator in the pathology of chronic kidney disease (CKD) (40), which could explain the above discrepancies. In the present meta-analysis, all included studies were adjusted for CKD, resulting in a CKD-independent effect assessment of the association between serum FGF-23 level and the risk of AF. Consistently, another prospective cohort study which enrolled 3,876 patients with mild-to-severe CKD reported that a 1-U increase in serum FGF23 level increased the risk of AF by 47% (10). The potential reasons for the discrepancies among these studies might be attributed to significant differences in the relevant risk factor profiles or the incidence of AF. Moreover, as an early biomarker for CKD, Klotho deficiency contributes to soft-tissue calcification in CKD, and Klotho was considered a co-receptor for FGF23 function (41). In addition, α-Klotho deficiency in CKD patients may exacerbate α-Klotho-independent cardiac toxicity of FGF23, thereby promoting the incidence of AF (42). However, a limited number of studies have detected the serum α-Klotho levels. Therefore, further studies are warranted to assess the role of α-Klotho in the association between serum FGF-23 level and the risk of AF, especially in patients with CKD.

### Potential mechanisms

Several potential mechanisms can explain the association between serum GDF-15 and FGF-23 levels and the risk of AF. GDF-15 is a non-specific indicator of cellular stress, inflammation, and biological aging (43). Experiments have shown that GDF-15 secretion increases after myocardial cells are stimulated. It enhances the proliferation of fibroblasts and may be involved in the progression of myocardial fibrosis (44). At the same time, GDF-15 blocks norepinephrine-induced myocardial hypertrophy activation through a new pathway of inhibition of epidermal growth factor receptors, which is closely related to atrial structural remodeling and atrial fibrosis (30, 45). GDF-15 may also play an essential role in atrial structural remodeling through collagen synthesis and transformation, thus participating in the occurrence of AF (30). In addition, GDF-15 is closely related to inflammation (46), which may stimulate an increase in the production of inflammatory factors such as IL-6. However, the causal relationship between AF and inflammation has not been clarified (47), and the inflammatory mechanism associated with GDF-15 still needs to be further explored.

FGF-23 plays a pivotal role in regulating mineral homeostasis and can promote myocardial remodeling and myocardial hypertrophy, causing endothelial dysfunction (8, 38). High serum levels of FGF-23 lead to dysregulation of the levels of calcium, phosphorus, and vitamin D in the body. Evidence suggests that elevated FGF-23 parallels decreased α-klotho and calcitriol levels. Reduced α-klotho levels may promote the aging of cardiomyocytes and is associated with the development of AF. As calcitriol is a key factor in regulating calcium and phosphorus metabolism, the imbalance of calcium ions in the body directly leads to cardiac electrical activity disorder, eventually resulting in AF (48). FGF-23 upregulation may also activate the renin-angiotensin-aldosterone system (RAAS), which plays a role in atrium remodeling and influences the hemodynamics of the kidneys (49), thereby indirectly affecting the cardiac function (50). The reninic effect of calcitriol also affects the RAAS system (48). Furthermore, studies have demonstrated that FGF-23 significantly activates the protein kinase C (PKC) signaling pathway, resulting in abnormal sodium channel conductance, affecting cardiac function, and disrupting the heart rate (51, 52). In addition, FGF-23 can also activate the TGF-β signaling pathway, leading to the activation of fibroblasts (53). During AF, the atrium undergoes fibrosis, resulting from abnormal collagen metabolism caused by FGF-23, such as type I and type III collagen imbalances; and collagen fiber alignment disorders can further lead to the occurrence of AF (53, 54). The roles of the inflammatory factor IL6 and TNF have also been reported, but the specific mechanisms have not been investigated thoroughly (48).

### Implications and further research

NT-pro BNP and inflammation play important roles in the occurrence and development of AF (17). No significant association between serum FGF-23 levels and the risk of AF was found in the subgroups without adjustment for NT-pro BNP and CRP levels in the categorical analysis, but only one study was included. In contrast, in the dose-dependent effect analysis, all studies were adjusted for NT-pro BNP/CRP, and positive results were observed. The above-mentioned results suggested that FGF-23 could increase the risk of AF irrespective of NT-pro BNP and CRP levels. However, additional studies are required to confirm our results.

To date, several risk models have been established to predict the incidence of AF in the general population (31–33). Recent studies explored the roles of biomarkers in improving the predictive abilities of AF risk scores. This study demonstrated the predictive value of serum FGF-23 levels in AF. Further studies are needed to investigate its predictive performance by including FGF-23 to the existing AF prediction scores.

Furthermore, although our study did not yield a positive correlation between GDF-15 and AF risk, GDF-15 has been applied as a biomarker for predicting the risk of cardiovascular disease (46, 55), and the prognosis of AF (56). According to several large cohort studies, the biomarker-based ABC (age, biomarker, and clinical history) score incorporating GDF-15 has demonstrated good predictive ability for embolization and bleeding events in AF patients (57–59). Hijazi et al. showed that the ABC bleeding score performed better than the HAS-BLED and ORBIT scores (60), suggesting a promising clinical application.

However, FGF-23 may be applied in predicting renal outcomes in diabetic nephropathy (61), assessing glomerular filtration rate, and identifying abnormalities associated with chronic kidney disease (62, 63). This implies that FGF-23 levels are affected by other diseases, which may interfere with the prediction of atrial fibrillation. Limited by the specificity and sensitivity of biomarkers in predicting AF, these two biomarkers are not yet routinely performed in many hospitals and laboratories, restricting their widespread use. We believe that a single biomarker alone is unlikely to meet all expectations and that a combination of other biomarkers and further exploration is required for clinical application.

### Study limitations

This was the first study that assessed the dose-dependent associations of FGF-23 and GDF-15 with the risk of AF. Nevertheless, several limitations of this study should be pointed out. Firstly, only a relatively small number of articles was included in this meta-analysis, and some articles used unconventional units of GDF-15 levels, and they were excluded (35). Due to the inherent flaws of observational studies, causality cannot be drawn. Secondly, a moderate or a high degree of heterogeneity was found in this meta-analysis. The heterogeneity might be partly due to differences in participants' characteristics, study design, and analysis methods. Heterogeneity decreased after removing Chen's study (31), suggesting the heterogeneity was due to regional differences. Furthermore, considering the limited number of included studies (*N* < 10), the meta-regression was not conducted to assess the source of heterogeneity according to the guidelines (64). Thirdly, although serum FGF-23 levels were elevated in patients with CKD, subgroup analyses stratified by CKD were not conducted due to data restrictions. Further studies are needed to elucidate the association of serum FGF23 levels with the risk of AF in patients with or without CKD. Finally, multiple other confounders, such as diabetes, hypertension, ischemic heart disease, and heart failure, were not included in the subgroup analysis due to data restriction. Further prospective studies are needed to independently assess the association of GDF-15/FGF-23 to the risk of AF.

## Conclusions

In summary, our study showed a positive linear association of serum FGF-23 levels with the risk of AF. No significant association between serum GDF-15 levels and the risk of AF was found. Further studies are needed to verify whether FGF-23 may be applied in predicting the risk of AF.

## Data availability statement

The datasets presented in this study can be found in online repositories. The names of the repository/repositories and accession number(s) can be found in the article/Supplementary material.

## Author contributions

XL contributed to the study concept, design, and revised the draft. ZT, TS, and SH performed the search strategy, contributed to database research, acquisition of data, and statistical analyses. All authors participated in data analysis, reviewed, and approved the final manuscript.

## Funding

This work was supported in part by the National Natural Science Foundation of China (Nos. 81760050, 81760048, and 82100347), the Jiangxi Provincial Natural Science Foundation for Youth Scientific Research (No. 20192ACBL21037), and the China Postdoctoral Science Foundation (2021M703724).

## Conflict of interest

The authors declare that the research was conducted in the absence of any commercial or financial relationships that could be construed as a potential conflict of interest.

## Publisher's note

All claims expressed in this article are solely those of the authors and do not necessarily represent those of their affiliated organizations, or those of the publisher, the editors and the reviewers. Any product that may be evaluated in this article, or claim that may be made by its manufacturer, is not guaranteed or endorsed by the publisher.
