# Supplementary material for: Relationship between serum growth differentiation factor 15, fibroblast growth factor-23 and risk of atrial fibrillation: A systematic review and meta-analysis
Source: Front Cardiovasc Med. 2022 Aug 4;9:899667. doi: 10.3389/fcvm.2022.899667 (PMC9386045; doi:10.3389/fcvm.2022.899667)

| **Section and Topic** | **Item #** | **Checklist item** | **Location where item is reported** |
| --- | --- | --- | --- |
| **TITLE** | | |  |
| Title | 1 | Identify the report as a systematic review. | 1 |
| **ABSTRACT** | | |  |
| Abstract | 2 | See the PRISMA 2020 for Abstracts checklist. | 3 |
| **INTRODUCTION** | | |  |
| Rationale | 3 | Describe the rationale for the review in the context of existing knowledge. | 3 |
| Objectives | 4 | Provide an explicit statement of the objective(s) or question(s) the review addresses. | 3 |
| **METHODS** | | |  |
| Eligibility criteria | 5 | Specify the inclusion and exclusion criteria for the review and how studies were grouped for the syntheses. | 4 |
| Information sources | 6 | Specify all databases, registers, websites, organisations, reference lists and other sources searched or consulted to identify studies. Specify the date when each source was last searched or consulted. | 5 |
| Search strategy | 7 | Present the full search strategies for all databases, registers and websites, including any filters and limits used. | 5 |
| Selection process | 8 | Specify the methods used to decide whether a study met the inclusion criteria of the review, including how many reviewers screened each record and each report retrieved, whether they worked independently, and if applicable, details of automation tools used in the process. | 5-6 |
| Data collection process | 9 | Specify the methods used to collect data from reports, including how many reviewers collected data from each report, whether they worked independently, any processes for obtaining or confirming data from study investigators, and if applicable, details of automation tools used in the process. | 6 |
| Data items | 10a | List and define all outcomes for which data were sought. Specify whether all results that were compatible with each outcome domain in each study were sought (e.g. for all measures, time points, analyses), and if not, the methods used to decide which results to collect. | 6 |
|  | 10b | List and define all other variables for which data were sought (e.g. participant and intervention characteristics, funding sources). Describe any assumptions made about any missing or unclear information. | 7 |
| Study risk of bias assessment | 11 | Specify the methods used to assess risk of bias in the included studies, including details of the tool(s) used, how many reviewers assessed each study and whether they worked independently, and if applicable, details of automation tools used in the process. | 6-7 |
| Effect measures | 12 | Specify for each outcome the effect measure(s) (e.g. risk ratio, mean difference) used in the synthesis or presentation of results. | 6 |
| Synthesis methods | 13a | Describe the processes used to decide which studies were eligible for each synthesis (e.g. tabulating the study intervention characteristics and comparing against the planned groups for each synthesis (item #5)). | 6 |
|  | 13b | Describe any methods required to prepare the data for presentation or synthesis, such as handling of missing summary statistics, or data conversions. | 6 |
|  | 13c | Describe any methods used to tabulate or visually display results of individual studies and syntheses. | 6 |
|  | 13d | Describe any methods used to synthesize results and provide a rationale for the choice(s). If meta-analysis was performed, describe the model(s), method(s) to identify the presence and extent of statistical heterogeneity, and software package(s) used. | 6 |
|  | 13e | Describe any methods used to explore possible causes of heterogeneity among study results (e.g. subgroup analysis, meta-regression). | 6 |
|  | 13f | Describe any sensitivity analyses conducted to assess robustness of the synthesized results. | 6 |
| Reporting bias assessment | 14 | Describe any methods used to assess risk of bias due to missing results in a synthesis (arising from reporting biases). | 7 |
| Certainty assessment | 15 | Describe any methods used to assess certainty (or confidence) in the body of evidence for an outcome. | 7 |
| **RESULTS** | | |  |
| Study selection | 16a | Describe the results of the search and selection process, from the number of records identified in the search to the number of studies included in the review, ideally using a flow diagram. | 7 |
|  | 16b | Cite studies that might appear to meet the inclusion criteria, but which were excluded, and explain why they were excluded. | 7 |
| Study characteristics | 17 | Cite each included study and present its characteristics. | 7-8 |
| Risk of bias in studies | 18 | Present assessments of risk of bias for each included study. | 7-8 |
| Results of individual studies | 19 | For all outcomes, present, for each study: (a) summary statistics for each group (where appropriate) and (b) an effect estimate and its precision (e.g. confidence/credible interval), ideally using structured tables or plots. | 8-9 |
| Results of syntheses | 20a | For each synthesis, briefly summarise the characteristics and risk of bias among contributing studies. | 8-9 |
|  | 20b | Present results of all statistical syntheses conducted. If meta-analysis was done, present for each the summary estimate and its precision (e.g. confidence/credible interval) and measures of statistical heterogeneity. If comparing groups, describe the direction of the effect. | 8-9 |
|  | 20c | Present results of all investigations of possible causes of heterogeneity among study results. | 8-9 |
|  | 20d | Present results of all sensitivity analyses conducted to assess the robustness of the synthesized results. | 8-9 |
| Reporting biases | 21 | Present assessments of risk of bias due to missing results (arising from reporting biases) for each synthesis assessed. | 8-9 |
| Certainty of evidence | 22 | Present assessments of certainty (or confidence) in the body of evidence for each outcome assessed. | 8-9 |
| **DISCUSSION** | | |  |
| Discussion | 23a | Provide a general interpretation of the results in the context of other evidence. | 9 |
|  | 23b | Discuss any limitations of the evidence included in the review. | 9-11 |
|  | 23c | Discuss any limitations of the review processes used. | 12 |
|  | 23d | Discuss implications of the results for practice, policy, and future research. | 12-13 |
| **OTHER INFORMATION** | | |  |
| Registration and protocol | 24a | Provide registration information for the review, including register name and registration number, or state that the review was not registered. | 13-14 |
|  | 24b | Indicate where the review protocol can be accessed, or state that a protocol was not prepared. | 13-14 |
|  | 24c | Describe and explain any amendments to information provided at registration or in the protocol. | 13-14 |
| Support | 25 | Describe sources of financial or non-financial support for the review, and the role of the funders or sponsors in the review. | 14 |
| Competing interests | 26 | Declare any competing interests of review authors. | 14 |
| Availability of data, code and other materials | 27 | Report which of the following are publicly available and where they can be found: template data collection forms; data extracted from included studies; data used for all analyses; analytic code; any other materials used in the review. | 14 |

*From:*  Page MJ, McKenzie JE, Bossuyt PM, Boutron I, Hoffmann TC, Mulrow CD, et al. The PRISMA 2020 statement: an updated guideline for reporting systematic reviews. BMJ 2021;372:n71. doi: 10.1136/bmj.n71

For more information, visit: <http://www.prisma-statement.org/>

**Table S2:** Detailed description of the search strategy

| **PubMed** | |
| --- | --- |
| GDF-15 | |
| #1 | growth differentiation factor 15 [MeSH Terms] |
| #2 | atrial fibrillation [MeSH Terms] |
| #3 | (Macrophage inhibitory cytokine 1) OR (Prostate differentiation factor) OR (GDF-15) |
| #4 | (Atrial Fibrillations) OR (Auricular Fibrillation) OR (Auricular Fibrillations) OR (Persistent Atrial Fibrillation) OR (Familial Atrial Fibrillation) OR (Paroxysmal Atrial Fibrillation) |
| #5 | #2 OR #4 |
| #6 | #1 OR #3 |
| #7 | #5 AND #6 |
| FGF-23 | |
| #1 | atrial fibrillation [MeSH Terms] |
| #2 | (Atrial Fibrillations) OR (Auricular Fibrillation) OR (Auricular Fibrillations) OR (Persistent Atrial Fibrillation) OR (Familial Atrial Fibrillation) OR (Paroxysmal Atrial Fibrillation) |
| #3 | fibroblast growth factor-23 |
| #4 | (Fgf23 protein) OR (fibroblast growth factor 23) OR (FGF-23 protein) OR (phosphatonin) OR (tumor-derived hypophophatemia inducing factor) |
| #5 | #3 OR #4 |
| #6 | #1 OR #2 |
| #7 | #5 AND #6 |
| **Embase** | |
| GDF-15 | |
| #1 | (Atrial Fibrillations) OR (Auricular Fibrillation) OR (Auricular Fibrillations) OR (Persistent Atrial Fibrillation) OR (Familial Atrial Fibrillation) OR (Paroxysmal Atrial Fibrillation) |
| #2 | (Macrophage inhibitory cytokine 1) OR (Prostate differentiation factor) OR (GDF-15) |
| #3 | atrial fibrillation |
| #4 | growth differentiation factor 15 |
| #5 | #2 OR #4 |
| #6 | #1 OR #3 |
| #7 | #5 AND #6 |
| FGF-23 | |
| #1 | atrial fibrillation |
| #2 | (Atrial Fibrillations) OR (Auricular Fibrillation) OR (Auricular Fibrillations) OR (Persistent Atrial Fibrillation) OR (Familial Atrial Fibrillation) OR (Paroxysmal Atrial Fibrillation) |
| #3 | fibroblast growth factor-23 |
| #4 | (Fgf23 protein) OR (fibroblast growth factor 23) OR (FGF-23 protein) OR (phosphatonin) OR (tumor-derived hypophophatemia inducing factor) |
| #5 | #3 OR #4 |
| #6 | #1 OR #2 |
| #7 | #5 AND #6 |
| **Cochrane** | |
| GDF-15 | |
| #1 | growth differentiation factor 15 [MeSH Terms] |
| #2 | atrial fibrillation [MeSH Terms] |
| #3 | GDF-15 |
| #4 | Macrophage inhibitory cytokine 1 |
| #5 | Prostate differentiation factor |
| #6 | persistent atrial fibrillation |
| #7 | paroxysmal atrial fibrillation |
| #8 | familial atrial fibrillation |
| #9 | atrial fibrillations |
| #10 | auricular fibrillations |
| #11 | auricular fibrillation |
| #12 | #1 OR #3 OR #4 OR #5 |
| #13 | #2 OR #6 OR#7 OR #8 OR#9 OR #10 OR #11 |
| #14 | #12 AND #13 |
| FGF-23 | |
| #1 | persistent atrial fibrillation |
| #2 | paroxysmal atrial fibrillation |
| #3 | familial atrial fibrillation |
| #4 | atrial fibrillations |
| #5 | auricular fibrillations |
| #6 | auricular fibrillation |
| #7 | fibroblast growth factor-23 |
| #8 | Fgf23 protein |
| #9 | fibroblast growth factor 23 |
| #10 | FGF-23 protein |
| #11 | phosphatonin |
| #12 | tumor-derived hypophophatemia inducing factor |
| #13 | atrial fibrillation [MeSH Terms] |
| #14 | fibroblast growth factor-23 [MeSH Terms] |
| #15 | #13 OR #1 OR #2 OR #3 OR #4 OR #5 OR #6 |
| #16 | #14 OR #7 OR #8 OR #9 OR #10 OR #11 OR #12 |
| #17 | #15 AND #16 |

**Table S3:** Studies excluded (n=31) with reasons

| **Studies excluded** | **Reasons** |
| --- | --- |
| Doulamis, 2019^1^ | Cross-section |
| Galenko , 2019^2^ | Cross-section |
| Hijazi,2017^3^ | Cross-section |
| Izumiya, 2014^4^ | Cross-section |
| Matusik, 2020^5^ | Cross-section |
| Rivera-Caravaca, 2020^6^ | Cross-section |
| Tancin Lambert, 2020^7^ | Cross-section |
| Arbault-Biton, 2020 ^8^ | Not appropriate classification: AF time |
| Bansal, 2020 ^9^ | Meta-analysis |
| Berg, 2019 ^10^ | Not the target outcome: major bleeding |
| Hongisto, 2019 ^11^ | Not the target outcome: cardiogenic shock |
| Montoro-Gafcia, 2012 ^12^ | Not the target exposure: hypertrophic cardiomyopathy |
| Tan, 2021 ^13^ | Not the target outcome: heart failure |
| Wallentin, 2014 ^14^ | Not the target outcome: mortality |
| Miyamura, 2015 ^15^ | Cross-section |
| Geach, 2014 ^16^ | Review |
| Batra, 2016 ^17^ | Review |
| Ketteler, 2014 ^18^ | Review |
| Ford, 2016 ^19^ | Animal experiment |
| Begg, 2016 ^20^ | Not appropriate classification: DCCV |
| Chuan, 2019 ^21^ | Not interest data |
| Sxcialla, 2015^22^ | Not the target outcome: cardiovascular disease |
| Patel, 2020 ^23^ | Not the target outcome: atherosclerosis |
| Papadopoulos, 2016 ^24^ | Not the target exposure: chronic renal failure |
| Disthabanchong, 2018 ^25^ | Not the target exposure: chronic kidney disease |
| Ment, 2016 ^26^ | Meta analysis |
| Nowak, 2013 ^27^ | Not the target exposure: hemodialysis |
| Dong, 2018 ^28^ | Molecular experiment |
| Chan, 2018^29^ | Not the target outcome: ventricular hypertrophy |
| Coloma, 2018^30^ | Not the target outcome: cardiomyocyte injury |
| Seiler, 2011 ^31^ | Not interest data |

1. Doulamis IP, Samanidis G, Tzani A, et al. Proteomic profile of patients with atrial fibrillation undergoing cardiac surgery. *Interactive Cardiovascular and Thoracic Surgery* 2019; 28: 94-101. Article. DOI: 10.1093/icvts/ivy210.

2. Galenko O, Jacobs V, Knight S, et al. Circulating Levels of Biomarkers of Cerebral Injury in Patients with Atrial Fibrillation. *The American journal of cardiology* 2019; 124: 1697-1700. 2019/10/03. DOI: 10.1016/j.amjcard.2019.08.027.

3. Hijazi Z, Oldgren J, Andersson U, et al. Growth-differentiation factor 15 and risk of major bleeding in atrial fibrillation: Insights from the Randomized Evaluation of Long-Term Anticoagulation Therapy (RE-LY) trial. *American heart journal* 2017; 190: 94-103. 2017/08/02. DOI: 10.1016/j.ahj.2017.06.001.

4. Izumiya Y, Hanatani S, Kimura Y, et al. Growth differentiation factor-15 is a useful prognostic marker in patients with heart failure with preserved ejection fraction. *Can J Cardiol* 2014; 30: 338-344. 2014/02/04. DOI: 10.1016/j.cjca.2013.12.010.

5. Matusik PT, Małecka B, Lelakowski J, et al. Association of NT-proBNP and GDF-15 with markers of a prothrombotic state in patients with atrial fibrillation off anticoagulation. *Clinical research in cardiology : official journal of the German Cardiac Society* 2020; 109: 426-434. 2019/07/08. DOI: 10.1007/s00392-019-01522-x.

6. Rivera-Caravaca JM, Vílchez JA, Rodríguez-Rojas C, et al. Influence of the matrix type over the concentration of GDF-15. *Journal of Investigative Medicine* 2020; 68: 1402-1404. Article. DOI: 10.1136/jim-2020-001351.

7. Tancin Lambert A, Kong XY, Ratajczak-Tretel B, et al. Biomarkers Associated with Atrial Fibrillation in Patients with Ischemic Stroke: A Pilot Study from the NOR-FIB Study. *Cerebrovascular diseases extra* 2020; 10: 11-20. 2020/02/07. DOI: 10.1159/000504529.

8. Arbault-Biton C, Chenevier-Gobeaux C, Legallois D, et al. Multiple biomarkers measurement to estimate the duration of atrial fibrillation. *Annals of Clinical Biochemistry* 2020. Article in Press. DOI: 10.1177/0004563220975171.

9. Bansal N, Zelnick LR, Soliman E, et al. Change in Cardiac Biomarkers and Risk of Incident Heart Failure and Atrial Fibrillation in CKD: The Chronic Renal Insufficiency Cohort (CRIC) Study. *American journal of kidney diseases : the official journal of the National Kidney Foundation* 2020. Article in Press. DOI: 10.1053/j.ajkd.2020.09.021.

10. Berg DD, Ruff CT, Jarolim P, et al. Performance of the ABC scores for assessing the risk of stroke or systemic embolism and bleeding in patients with atrial fibrillation in ENGAGE AF-TIMI 48. *Circulation* 2019; 139: 760-771. Article. DOI: 10.1161/CIRCULATIONAHA.118.038312.

11. Hongisto M, Kataja A, Tarvasmäki T, et al. Levels of Growth Differentiation Factor 15 and Early Mortality Risk Stratification in Cardiogenic Shock. *Journal of Cardiac Failure* 2019; 25: 894-901. Article. DOI: 10.1016/j.cardfail.2019.07.003.

12. Montoro-García S, Hernández-Romero D, Jover E, et al. Growth differentiation factor-15, a novel biomarker related with disease severity in patients with hypertrophic cardiomyopathy. *European journal of internal medicine* 2012; 23: 169-174. 2012/01/31. DOI: 10.1016/j.ejim.2011.08.022.

13. Tan ESJ, Chan SP, Liew OW, et al. Atrial Fibrillation and the Prognostic Performance of Biomarkers in Heart Failure. *Clinical chemistry* 2021; 67: 216-226. Article. DOI: 10.1093/clinchem/hvaa287.

14. Wallentin L, Hijazi Z, Andersson U, et al. Growth differentiation factor 15, a marker of oxidative stress and inflammation, for risk assessment in patients with atrial fibrillation: Insights from the Apixaban for reduction in stroke and other thromboembolic events in atrial fibrillation (ARISTOTLE) trial. *Circulation* 2014; 130: 1847-1858. Article. DOI: 10.1161/CIRCULATIONAHA.114.011204.

15. Miyamura M, Fujita SI, Morita H, et al. Circulating fibroblast growth factor 23 has a U-shaped association with atrial fibrillation prevalence. *Circulation Journal* 2015; 79: 1742-1748. Article. DOI: 10.1253/circj.CJ-15-0413.

16. Geach T. Atrial fibrillation: FGF-23 associated with incident AF - A link with CKD? *Nature Reviews Cardiology* 2014; 11: 436. Note. DOI: 10.1038/nrcardio.2014.95.

17. Batra J, Buttar RS, Kaur P, et al. FGF-23 and cardiovascular disease: Review of literature. *Current Opinion in Endocrinology, Diabetes and Obesity* 2016; 23: 423-429. Review. DOI: 10.1097/MED.0000000000000294.

18. Ketteler M and Biggar PH. FGF23: More a matter of the heart than of the vessels? *Nephrology Dialysis Transplantation* 2014; 29: 1987-1988. Article. DOI: 10.1093/ndt/gfu276.

19. Ford K, Slavic S, Zeitz U, et al. Lack of fibroblast growth factor-23 (FGF23) preserves cardiac function in a murine model of acute myocardial infarction. *Heart* 2016; 102: A131. Conference Abstract. DOI: 10.1136/heartjnl-2016-309890.193.

20. Begg GA, Lip GYH, Plein S, et al. Circulating biomarkers of fibrosis and cardioversion of atrial fibrillation: A prospective, controlled cohort study. *Clinical Biochemistry* 2016. Article in Press. DOI: 10.1016/j.clinbiochem.2016.09.008.

21. Chua W, Purmah Y, Cardoso VR, et al. Data-driven discovery and validation of circulating blood-based biomarkers associated with prevalent atrial fibrillation. *Eur Heart J* 2019; 40: 1268-1276. 2019/01/08. DOI: 10.1093/eurheartj/ehy815.

22. Scialla JJ. Epidemiologic insights on the role of fibroblast growth factor 23 in cardiovascular disease. *Curr Opin Nephrol Hypertens* 2015; 24: 260-267. 2015/06/13. DOI: 10.1097/mnh.0000000000000123.

23. Patel RB, Ning H, de Boer IH, et al. Fibroblast Growth Factor 23 and Long-Term Cardiac Function: The Multi-Ethnic Study of Atherosclerosis. *Circ Cardiovasc Imaging* 2020; 13: e011925. 2020/11/10. DOI: 10.1161/circimaging.120.011925.

24. Papadopoulos C, Kassimatis E, Sampani E, et al. Klotho deficiency and high FGF23 plasma levels are associated with left atrial mechanical dysfunction in patients with chronic renal failure and paroxysmal atrial fibrillation history. *European Heart Journal* 2016; 37: 858. Conference Abstract. DOI: 10.1093/eurheartj/ehw433.

25. Disthabanchong S. Phosphate and Cardiovascular Disease beyond Chronic Kidney Disease and Vascular Calcification. *Int J Nephrol* 2018; 2018: 3162806. 2018/06/01. DOI: 10.1155/2018/3162806.

26. Meng L, Yang Y, Zhang Z, et al. Predictive value of circulating fibroblast growth factor-23 on atrial fibrillation: A meta-analysis. *Int J Cardiol* 2016; 210: 68-71. 2016/03/05. DOI: 10.1016/j.ijcard.2016.02.100.

27. Nowak A, Friedrich B, Artunc F, et al. Prognostic value and link to atrial fibrillation of circulating klotho and FGF23 in hemodialysis patients. *Nephrology Dialysis Transplantation* 2013; 28: i459. Conference Abstract. DOI: 10.1093/ndt/gft147.

28. Dong Q, Tang Y, Wang W, et al. Relationship between FGF23/FGFR4 expression in atrial tissue and atrial fibrosis in patients with atrial fibrillation. *National Medical Journal of China* 2018; 98: 1003-1007. Article. DOI: 10.3760/cma.j.issn.0376-2491.2018.13.010.

29. Chan CT, Kaysen GA, Beck GJ, et al. Changes in Biomarker Profile and Left Ventricular Hypertrophy Regression: Results from the Frequent Hemodialysis Network Trials. *American Journal of Nephrology* 2018; 47: 208-217. Article. DOI: 10.1159/000488003.

30. Coloma A, Azcarate P, Valdivielso JM, et al. Fibroblast growth factor 23 is associated with biomarkers of cardiomyocyte stress/injury in patients with CKD. *Journal of the American Society of Nephrology* 2018; 29: 514. Conference Abstract.

31. Seiler S, Cremers B, Rebling NM, et al. The phosphatonin fibroblast growth factor 23 links calcium-phosphate metabolism with left-ventricular dysfunction and atrial fibrillation. *Eur Heart J* 2011; 32: 2688-2696. 2011/07/08. DOI: 10.1093/eurheartj/ehr215.

**Table S4**. Quality assessment of included studies

| Author  (Publication Year) | Newcastle-Ottawa Scale | | | | | | | | | |
| --- | --- | --- | --- | --- | --- | --- | --- | --- | --- | --- |
|  | Selection | | | Comparability | | | Outcome | | | Total |
|  | a | b | c | d | e | f | g | h | i |  |
| Bening, 2019, | 1 | 1 | 1 | 1 | 1 | 1 | 1 | 0 | 1 | 8 |
| Santema, 2019, | 1 | 1 | 1 | 1 | 1 | 1 | 1 | 0 | 0 | 7 |
| Svennberg, 2016, | 1 | 1 | 1 | 0 | 1 | 1 | 1 | 1 | 1 | 8 |
| Smit,2011 | 1 | 1 | 1 | 1 | 1 | 1 | 1 | 0 | 1 | 8 |
| Shao, 2014 | 1 | 1 | 0 | 1 | 1 | 1 | 1 | 1 | 0 | 7 |
| Wei,2020 | 1 | 1 | 1 | 1 | 1 | 1 | 1 | 1 | 1 | 9 |
| Rienstra,2014 | 1 | 1 | 1 | 1 | 1 | 1 | 1 | 1 | 1 | 9 |
| Montealegre, 2019 | 1 | 1 | 1 | 0 | 1 | 1 | 1 | 1 | 0 | 7 |
| Olivier Bouchot, 2015 | 1 | 1 | 1 | 0 | 1 | 1 | 1 | 1 | 1 | 8 |
| Maan, 2016 | 1 | 1 | 1 | 1 | 1 | 1 | 1 | 1 | 0 | 8 |
| Chen, 2020 | 1 | 1 | 1 | 1 | 0 | 0 | 1 | 1 | 0 | 7 |
| Mizia-Stec, 2018 | 1 | 1 | 1 | 1 | 1 | 1 | 1 | 1 | 0 | 8 |
| Alonso, 2014 | 1 | 1 | 1 | 1 | 1 | 1 | 1 | 1 | 0 | 8 |
| Mathew, 2014 | 1 | 1 | 1 | 1 | 1 | 1 | 1 | 1 | 1 | 9 |
| Mehta, 2016 | 1 | 1 | 1 | 1 | 1 | 1 | 1 | 1 | 0 | 8 |

1. Representativeness of the exposed cohort.
2. Selection of the non-exposed cohort.
3. Ascertainment of exposure.
4. Demonstration that outcome of interest was not present at start of study.
5. Comparability of cohorts on the basis of the design or analysis (adjusted for age).
6. Comparability of cohorts on the basis of the design or analysis (adjusted for any other factor).
7. Assessment of outcome.
8. Was follow-up long enough for outcomes to occur. (>5 years for new on-set, 1 years for AF recurrence).
9. Adequacy of follow-up of cohorts.

**Figure S1** Subgroups analysis of GDF-15/FGF-23 and AF stratified by adjustments


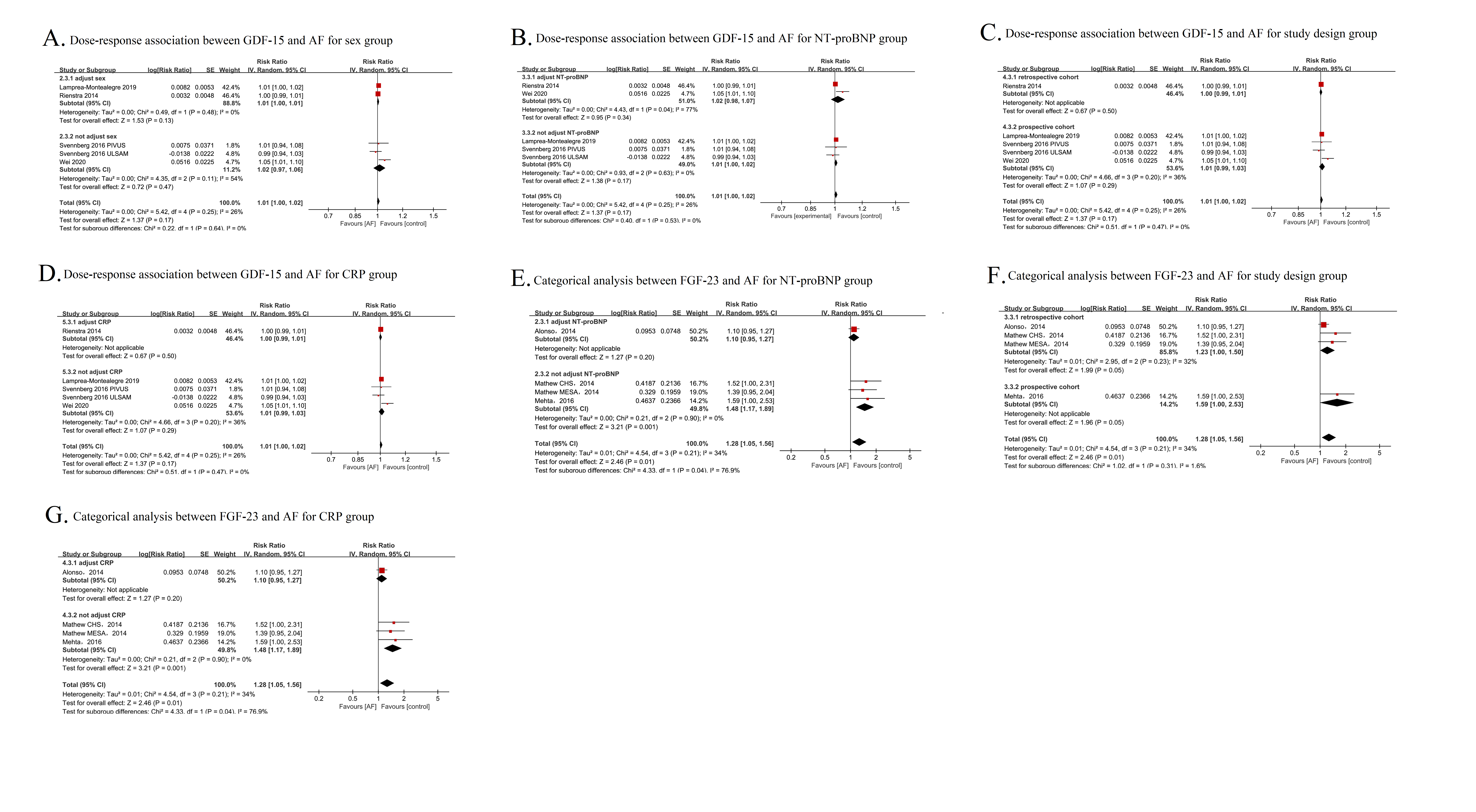

Supplement: Supplementary file 1 [file Data_Sheet_1.docx]
